# “Oil-soluble” reversed lipid nanoparticles for oral insulin delivery

**DOI:** 10.1186/s12951-020-00657-8

**Published:** 2020-07-17

**Authors:** Tao Wang, Liao Shen, Yadan Zhang, Haiyan Li, Yongan Wang, Dongqin Quan

**Affiliations:** State Key Laboratory of Toxicology and Medical Countermeasure, Beijing Institute of Pharmacology and Toxicology, Beijing, 100850 China

**Keywords:** Insulin, “Oil-soluble” reversed lipid nanoparticles, Oral absorption, Nanocarriers, Phospholipid

## Abstract

**Background:**

In this study, we aimed to design a novel oral insulin delivery system, named “oil-soluble” reversed lipid nanoparticles (ORLN), in which a hydrophilic insulin molecule is encapsulated by a phospholipid (PC) shell and dissolved in oil to prevent the enzymatic degradation of insulin. ORLN was characterized by transmission electron microscopy and dynamic light scattering.

**Results:**

In vitro enzymatic stability studies showed higher concentrations of insulin in cells incubated with ORLN-encapsulated insulin than in those incubated with free insulin solution in artificial intestinal fluid (pH 6.5). The protective effect of ORLN was attributed to its special release behavior and the formulation of the PC shell and oil barrier. Furthermore, an in vivo oral efficacy study confirmed that blood glucose levels were markedly decreased after ORLN administration in both healthy and diabetic mice. In vivo pharmacokinetic results showed that the bioavailability of ORLN-conjugated insulin was approximately 28.7% relative to that of the group subcutaneously administered with an aqueous solution of insulin, indicating enhanced oral absorption.

**Conclusions:**

In summary, the ORLN system developed here shows promise as a nanocarrier for improving the oral absorption of insulin.

## Background

Drug delivery via the oral route is preferred over other strategies, owing to high patient compliance and the ease of home-based drug ingestion [1, 2]. However, because of the low permeability and poor enzymatic stability of large molecules in the gastrointestinal (GI) tract, the oral route is usually not appropriate when proteins/peptides are directly delivered in a free form. The effective oral administration of protein-based drugs remains a challenge [3]. Insulin, the first protein-based drug synthesized for human use, has been used for the treatment of diabetes since 1921. Insulin is a 51-polypeptide hormone comprising an A chain (21 amino acids) and B chain (30 amino acids) linked by two disulfide bonds [4]. Its large size and hydrophilic nature hinder its permeation through absorption barriers. To date, most major commercial formulations of insulin are administered by subcutaneous injection. This conventional route of protein delivery may lead to poor patient compliance because of inconvenience and pain. To improve patient compliance, the oral route is considered a better alternative. In addition, the oral delivery of insulin is advantageous because it closely imitates the physiological behavior of endogenous insulin [2, 5]. Around 80% of oral/pancreatic insulin is cleared in the liver, while the rest reaches systemic circulation. Oral administration can thus avoid subcutaneous injection-related hyperinsulinemia. Several approaches have been adopted in recent decades to improve the oral bioavailability of insulin and other proteins/peptides [2, 6, 7]. Nevertheless, the development of a commercial oral formulation for insulin remains a challenge because no strategies have been able to successfully overcome both physicochemical drawbacks (molecular size, stability, and high hydrophilicity) and biological barriers (proteolysis in the stomach, poor permeation, and membrane efflux) [8, 9].

Recently, several products have completed or are currently undergoing phase II or phase III clinical trials, including oral insulin capsules (NIDDK; NIH, Bethesda, MD, USA), rH-insulin crystals (Technische Universität München, Munich, Germany), oral ORMD-0801 (Oramed, Jerusalem, Israel), Oshadi Icp (Oshadi Drug Administration, Rehovot, Israel), IN-105 (Biocon, Bangalore, India), insulin 338 (GIPET1), insulin glargine (Novo Nordisk, Bagsværd, Denmark), and an oral formulation of insulin (Nutrinia, Ramat Gan, Israel) [3, 10, 11]. Most approaches for oral insulin administration have focused on structural modifications, absorption and penetration enhancers, complicated carriers (nanoparticles, polymer micelles, liposomes), or enzyme inhibitors. Although these products have marginally improved the oral bioavailability of insulin, they require complicated formulations and excessive bioactive additives, which result in undesirable effects, increased manufacturing costs, and a high risk of drug development. Notably, Banerjee et al. [2] developed an ionic liquid (IL)-based oral formulation of insulin (insulin-CAGE). They reported an unprecedented improvement in the oral bioavailability of insulin. The oral bioavailability of 5 U/kg IJ insulin-CAGE was found to be 51% higher than that of 2 U/kg subcutaneous injection. Nonetheless, IL preparations have their own drawbacks, such as potential long-term toxicity, negative effects on the GI tract, and low biocompatibility. Hence, there has been increasing interest in drug delivery research to develop oral carriers of insulin showing both high efficacy and safety.

Our recent study reported a technique to encapsulate and “dissolve” water-soluble chemotherapeutic agents into vegetable oil by forming “oil-soluble” reversed lipid nanoparticles (ORLN) [12, 13]. This carrier can either decrease the intestinal hydrolytic degradation of topotecan (TPT) via protection by both a phospholipid (PC) shell and oil medium, or improve the oral absorption of TPT by enhancing the intestinal lymphatic transport.

In the present study, we designed an oral insulin formulation based on the ORLN system, in which amphipathic PC molecules could self-assemble to construct an internal polar pool for insulin molecules, with non-polar tails radiating to the outer oil phase to form ORLN. ORLN-insulin dispersed in medium-chain triglyceride (MCT) was prepared as an oral formulation to evaluate its efficacy and stability both in vitro and in vivo.

## Materials and methods

### Materials

Peptide recombinant human insulin (PHI) was kindly provided as a gift from the Department of Drug synthesis, Beijing Institute of Pharmacology and Toxicology (China). MCT was supplied by Gattefossé (Lyon, France). Soya phosphatidyl choline (LIPOID E80) was purchased from Lipoid GmbH (Ludwigshafen, Germany). High-performance liquid chromatography (HPLC)-grade acetonitrile and methanol were purchased from Thermo Fisher Scientific (Waltham, MA, USA). Human insulin enzyme-linked immunosorbent assay (ELISA) quantitative kit was purchased from Boster Biological Technology (Pleasanton, CA, USA). Simulated Intestinal Fluid Powder (SIF, pH 6.5) was purchased from Biorelevant (London, UK).

### Preparation of ORLN conjugated with insulin

**Fig. 1:**
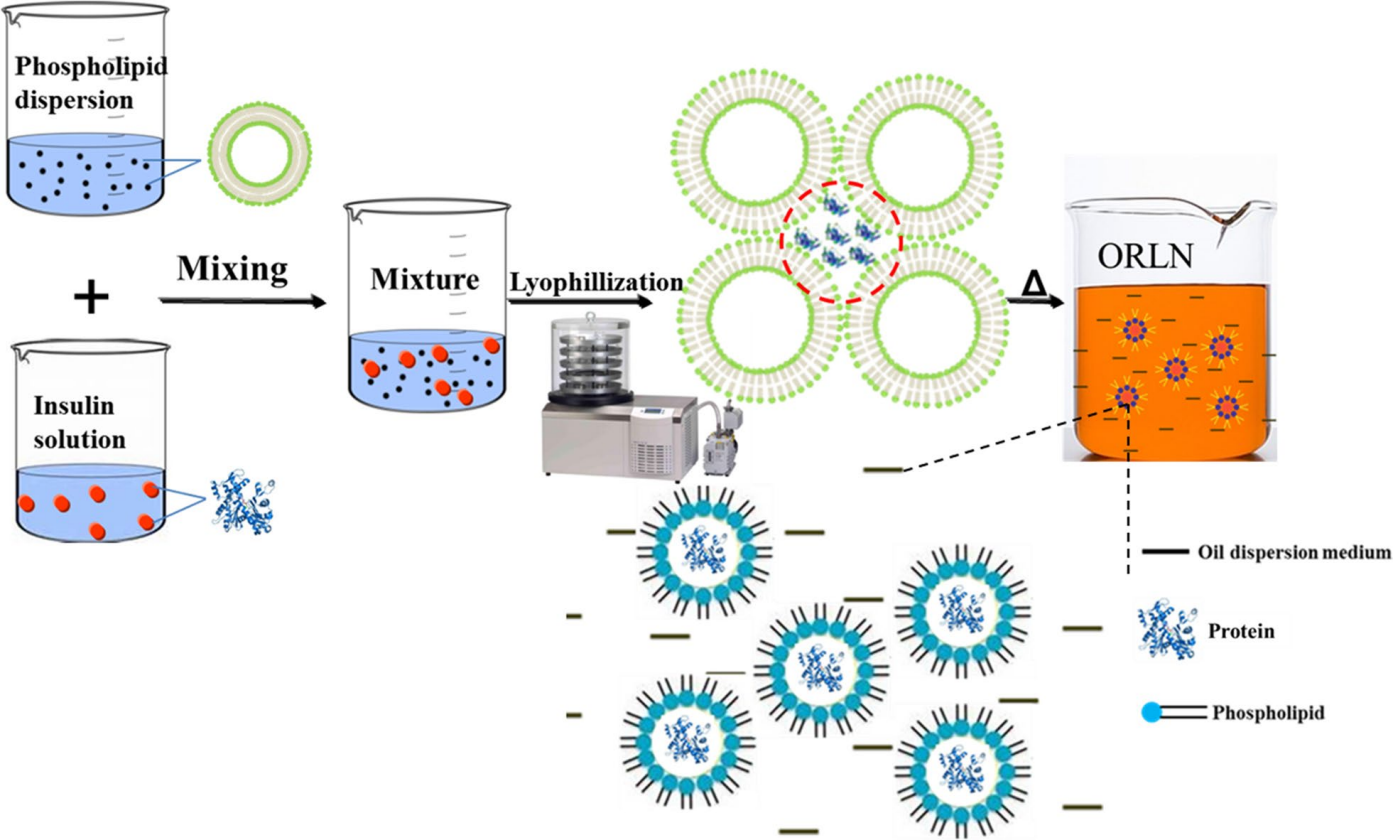
Preparation and structure of “oil-soluble” reversed lipid nanoparticle (ORLN)-insulin system

The oral formulations of insulin-conjugated ORLN (ORLN-PHI) were prepared through a two-step method (Fig. 1). First, small empty unilamellar liposomes were obtained by probe sonication as follows: 0.01–0.05 g lipoid S100 was dissolved in 15 mL chloroform, the solvent was evaporated by a rotary evaporator, and the dispersion was obtained by adding 10 mL pure water and shaking vigorously. The dispersion was sonicated using a probe-type sonicator at 200 W for 20 min and filtered through a 0.2-μm membrane (Millipore, Billerica, MA, USA). The PHI solution was prepared by dissolving 1.0 mg PHI in 10 mL acidic water. Then, a 2-mL mixture of the two solutions described above was added to a 7-mL vial at a ratio of 1:1 (v/v) and lyophilized in a freeze drier for 48 h (FD-1; Beijing SiHuan Technology Company, Beijing, China) to remove all water. Lyophilization was performed under the following conditions: freezing at − 50 °C for 4 h, primary at − 45 °C to − 10 °C for 20 h, secondary at − 10 °C to 20 °C for 15 h, and maintaining at 20 °C for 9 h. Finally, oral formulations of ORLN-PHI with different PC contents were obtained by dissolving the lyophilized dry cake in 0.5 mL MCT. The resulting formulation contained PHI at a concentration of 0.2 mg/mL.

### Physical characterization

#### Dynamic light scattering

The mean hydrodynamic diameter and size distribution of ORLN-PHI were determined by dynamic light scattering using Zetasizer (Nano ZS 90, Malvern Panalytical, Malvern, UK). The samples were obtained by diluting 0.1 mL ORLN-PHI in MCT to a total volume of 10 mL.

#### Transmission electron microcopy

The morphology of ORLN-PHI was observed by transmission electron microcopy (TEM). Before observation, 0.1 mL ORLN-PHI was diluted in 50 mL n-heptane. Then, 10 μL of the resulting solution was dropped on a cropper grid with carbon film, followed by the addition of 10 μL phosphotungstic acid solution. Excess fluid was removed using filter paper.

### In vivo oral efficacy of ORLN-PHI

The in vivo oral efficacy of ORLN-PHI was determined in both diabetic and healthy male Kunming mice. All animal experiments were approved by the Beijing Institute of Pharmacology and Toxicology ethics committee and were in accordance with the Chinese laboratory animal welfare and ethics guidelines. Type I diabetes was induced in Kunming mice by streptozotocin according to a previous method [15]. Both diabetic and healthy mice weighing 20 ± 2 g were fasted for 12 h, but allowed access to water prior to and during the experiments. Twenty healthy mice and fifteen diabetic mice were randomly divided into three groups each. Each group of mice (both healthy and diabetic) was given the same dose (600 μg/kg) of PHI by gavage in the form of ORLN-PHI or aqueous PHI solution, or by subcutaneous injection of PHI aqueous solution. A series of doses of ORLN-PHI were administered to healthy mice to determine the dose–response relationship. Blood glucose levels were measured using a commercial glucose meter at the predetermined time point until glucose levels increased again. The results were plotted as the percent change in blood level versus time.

### Pharmacokinetic studies

Adult diabetic male Wistar rats induced by streptozotocin as previously described [14] were used for pharmacokinetic studies. Eighteen model animals with blood glucose levels > 25 mmol/L were selected and fasted overnight with free access to water. The next day, the rats were randomly divided into three groups of six rats each. Group I was subcutaneously injected with PHI aqueous solution containing 12 μg PHI. Group II and group III were orally administered with either ORLN-PHI or free PHI solution containing the same concentration of PHI. Blood samples were collected from the tail vein of rats at different times after dosing. Approximately 250 μL blood samples were drawn into heparin-treated centrifuge tubes and centrifuged at 16,161×*g* for 2.5 min. The plasma samples were collected and stored at -20 °C until analysis. Plasma PHI levels were determined by ELISA (Boster). The results were plotted as plasma PHI level vs. time, and the pharmacokinetic parameters were calculated from the graph. The relative bioavailability was calculated as follows:

$$Fr\% = \left[ {\frac{{D_{oral} \times Dose_{sub} }}{{D_{sub} \times Dose_{oral} }}} \right] \times 100$$

where *D*_*oral*_ is area under concentration time curve of plasma PHI after oral dosing PRLN-PHI formulations, *D*_*sub*_ is area under concentration time curve of plasma PHI after subcutaneous injection PHI aqueous solution. *Dose*_*oral*_ and *Dose*_*sub*_ are the doses of two test formulations described above respectively.

### Mechanism of action of ORLN-PHI on the enhancement of oral insulin delivery

#### In vitro release

The in vitro release of insulin from ORLN-PHI with different PC contents was measured to study the effect of PC on the release behavior of ORLN-PHI. A specific concentration of sample corresponding to 50 μg PHI was placed in 10 mL SIF medium under dynamic conditions (650 rpm, 37 ± 0.1 °C). Then, 0.2-mL aliquots of medium were withdrawn and replaced with equal volumes of fresh medium at 15, 30, 60, 90, and 120 min. The supernatants were obtained by centrifugation at 16,161×*g* for 30 s to remove oil drops. The supernatant (100 μL) was subjected to HPLC for PHI content determination. HPLC analysis was performed using the Agilent HPLC system (Agilent Technologies, Santa Clara, CA, USA) equipped with C_4_ column (250 × 4.6 mm, 5 μm; Kromasil, Amsterdam, The Netherlands). The mobile phase comprised 0.1% trifluoroacetic acid (TFA) in water for phase A and 0.09% TFA in acetonitrile for phase B. Each sample was run for 20 min at a flow rate of 1.0 mL/min using a gradient procedure where the amount of organic phase was increased from 10 to 70% over 15 min. The detection wavelength was 214 nm.

#### In vitro enzymatic stability

The experimental medium was prepared by adding 0.25 g pancreatin to 25 mL SIF. ORLN-PHI formulations with different PC contents were placed in the experimental medium under the same dynamic conditions described above (“In vitro release” section). At each predetermined time point (15, 30, 60, 90, and 120 min), 0.2 mL medium was removed and 10% TFA aqueous solution was added to terminate the reaction. The samples were vortexed for 1 min and centrifuged at 16,161×*g* for 30 s to obtain the supernatant. The supernatant (100 μL) was analyzed by HPLC as described in “In vitro release” section.

#### In vitro transport assay of insulin

A rapid 3-day Caco-2 growth system was utilized for transport experiments. Briefly, 500 µL cell solution containing basal seeding medium (BSM) supplemented with MITO + serum extender was seeded at a density of 4 × 10^5^ cells/mL on the apical side of polycarbonate film (PCF) membranes in 12-well transwell plates, while 1000 µL cell-free BSM was added to the basolateral side. The Caco-2 monolayers were incubated as previously reported with slight modification [14]. The transport study was performed when the transepithelial electrical resistance (TEER) reached above 200 Ω cm^2^.

Before the start of the experiment, existing medium in the transwell plates was replaced with Dulbecco’s modified Eagle’s medium (DMEM) devoid of phenol red, fetal bovine serum (FBS), and penicillin/streptomycin (P/S) in both the apical (200 µL) and basolateral sides (600 µL). After 30 min incubation, the medium in the apical side was replaced with 200 µL 50 µg/mL fluorescein isothiocyanate (FITC)-conjugated insulin in free form or encapsulated in ORLN with 1, 5, or 50 mg PC shells dispersed in DMEM without phenol red, FBS, and P/S at dynamic conditions (100 rpm, 37 ± 0.1 °C, 5% CO_2_). Then, 100-µL aliquots were withdrawn from the basolateral side and replaced with equal volumes of fresh DMEM at 0, 1, 2, 3, 4, and 5 h. Aliquots were determined by a plate reader (Infinite M1000; Tecan, Männedorf, Switzerland) to determine the concentration of FITC-insulin at excitation and emission wavelengths of 495 and 520 nm, respectively. TEER was measured at each time point. At the end of the investigation, the transwell plates used for the FITC-insulin transport study were washed twice with Hanks’ Balanced Salt Solution (HBSS) followed by the addition of 100 µL 4% paraformaldehyde, and washed twice with phosphate-buffered saline. The transwell membranes were gently placed on glass slides and observed under a fluorescence inverted microscope (Fluoview 1000; Olympus, Tokyo, Japan) at 40× magnification.

### Statistical analysis

All data are presented as the mean ± standard deviation (SD), and the analysis of differences between more than two groups was performed by one-way analysis of variance (ANOVA). A value of p < 0.05 was considered statistically significant.

## Results

### Preparation and characterization of ORLN-PHI

**Fig. 2:**
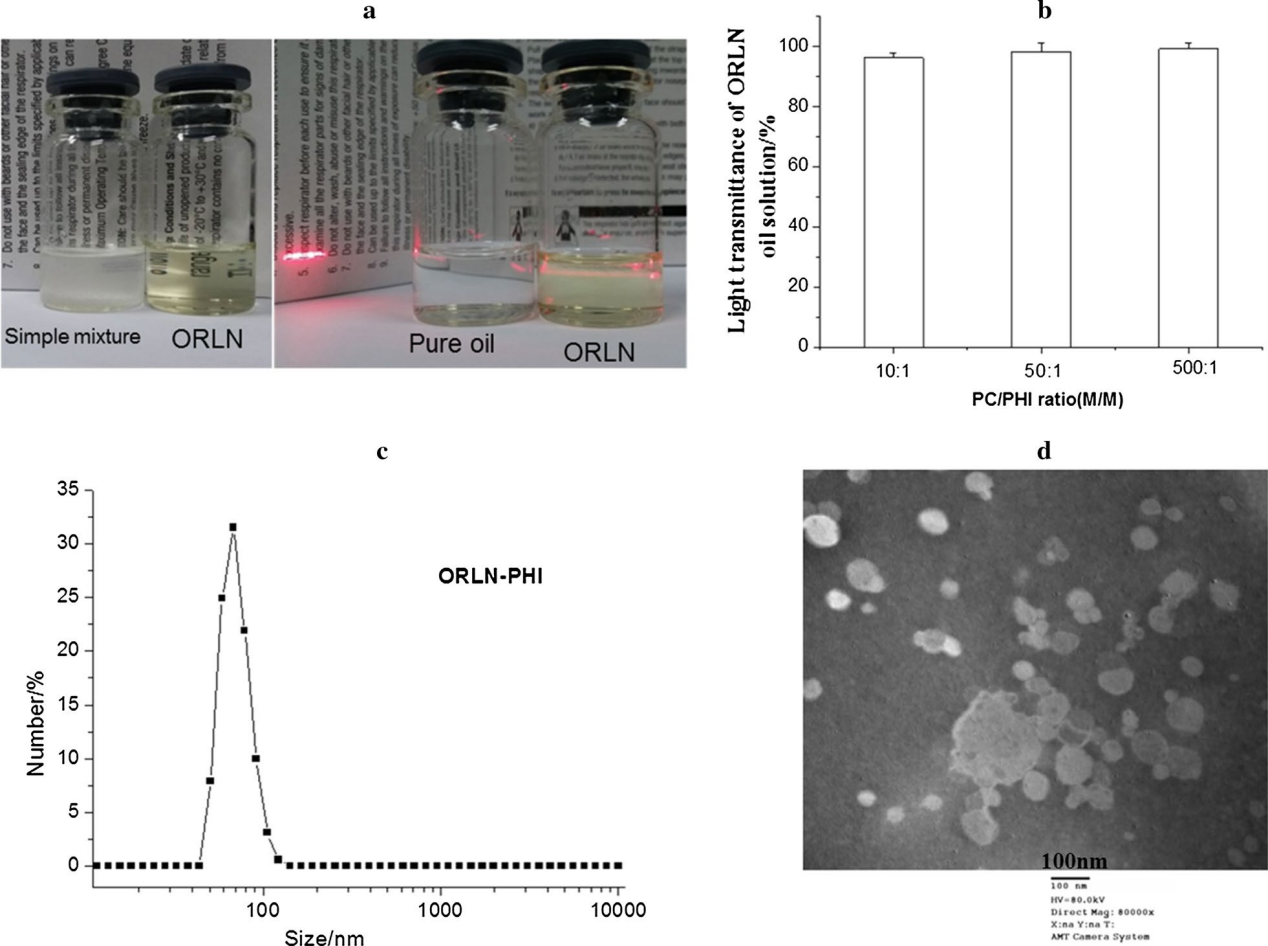
**a** The ORLN-peptide recombinant human insulin (PHI) formulations. Right: the Tyndall phenomenon in a pure oil solution. Left: the ORLN-PHI solution was transparent and distinct from a simple mixture of pure oil and insulin powder. **b** Light transmittance of ORLN-PHI formulations with different phospholipid (PC)/PHI ratios. **c** Size distribution of ORLN-PHI (PC/PHI ratio 500:1) by dynamic light scattering. **d** Transmission electron micrograph of ORLN-PHI (PC/PHI ratio 500:1)

Our previous study showed that water-soluble drugs can be efficiently encapsulated into ORLN using a simple three-step technique [12, 13]. In this study, 200 μg/mL ORLN-PHI oil solution was successfully formed by dissolving the lyophilized dry cake in MCT. Unlike the simple mixture of PHI and MCT, ORLN-PHI solution had a transparent appearance with light yellow color (Fig. 2a). The transmittance of ORLN-PHI solutions with different PC contents at 600 nm was more than 95% (Fig. 2b). As shown in Fig. 2a, ORLN-PHI solution showed a clear Tyndall phenomenon, indicating the presence of a nanostructure.

The particle size and distribution of ORLN-PHI are presented in Table 1 and Fig. 2c. ORLN-PHI had a small size, PC was homogeneously distributed. Increased PC content resulted in a slight increase in particle size. The morphology of ORLN-PHI was confirmed using TEM (Fig. 2d). The nanoparticles were spherical or ellipsoid in shape, and most of them were around 100 nm in size.

**Table 1:** Particle size of ORLN-PHI solutions with different PC/PHI ratios

PC/PHI ratio (w/w)	Particle size (nm)
10:1	75.3 ± 2.5
50:1	72.2 ± 7.6
500:1	70.8 ± 5.3

### In vivo oral efficacy of ORLN-PHI

**Fig. 3:**
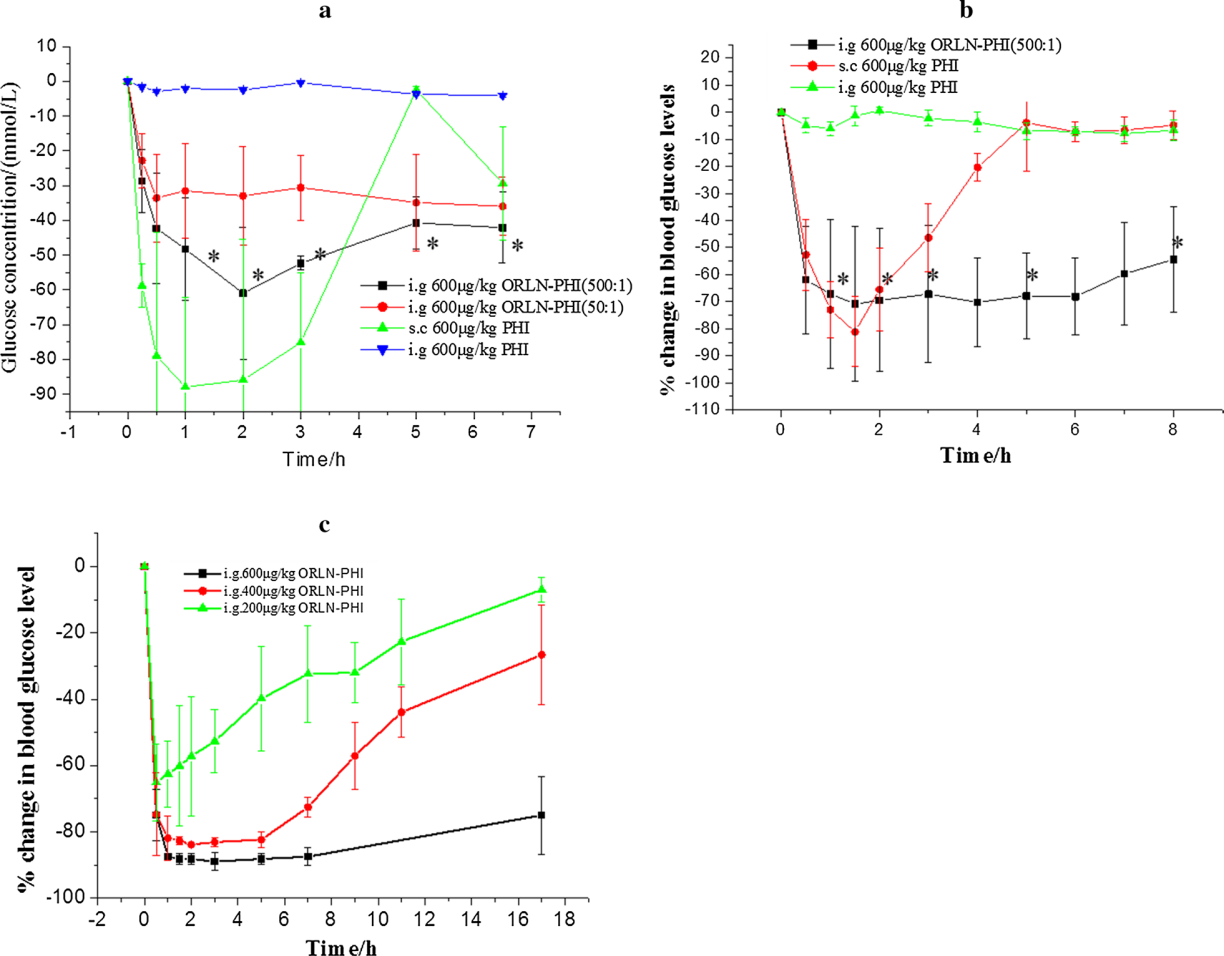
Efficacy of ORLN-PHI in lowering blood glucose levels following gavage administration in both healthy and diabetic mice. **a** Blood glucose-lowering efficacy of various formulations compared with initial levels in healthy mice. **b** Blood glucose-lowering efficacy of various formulations compared with initial levels in diabetic mice. **c** Blood glucose-lowering efficacy of various doses of ORLN-PHI (PC/PHI ratio 500:1) in healthy mice. All data are represented as the mean ± SE (n = 6). *p < 0.05

To examine the effect of ORLN on the oral absorption of PHI, its oral efficacy was determined by administration of a single dose of PHI to both healthy and diabetic mice. In addition, free PHI aqueous solution was administered orally as a control. The average blood glucose level over time in mice treated with ORLN-PHI or free PHI is shown in Fig. 3. Diabetic mice treated with 600 μg/kg ORLN-PHI demonstrated a rapid 70.2% decrease in blood glucose level within 2 h. This hypoglycemic effect was sustained till the end of the experiment. Subcutaneous administration of the same dose of free PHI led to a sharp 81.1% drop in blood glucose level after 2 h, which increased rapidly to baseline levels at 4 h. In the diabetic mice orally administered with the same dose of free PHI solution, no significant change in blood glucose was observed. Similar results were obtained in healthy mice. After oral administration, ORLN-PHI demonstrated a significant sustained hypoglycemic effect until the end of the study, unlike the subcutaneous administration of PHI. Oral administration of free PHI did not demonstrate any biological effects. A significant difference in the efficacy between ORLN-PHI and free PHI was observed. To confirm the enhancement of PHI delivery by ORLN system, a series of doses of ORLN-PHI formulations were administered to healthy mice. The results indicated that the hypoglycemic efficacy of ORLN-PHI was dose-dependent. Mice administered different doses of ORLN-PHI showed a clear drop in blood glucose levels during the testing period. Furthermore, the oral efficacy of ORLN-PHI was maintained for 17 h for all three doses of ORLN-PHI, indicating that ORLN provided an excellent oral sustained release preparation for the delivery of insulin once daily.

### Pharmacokinetics studies in rats

**Fig. 4:**
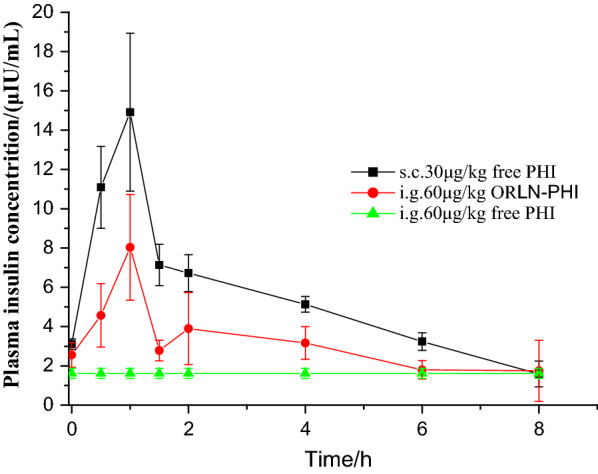
Mean plasma insulin concentrations over time after administration of ORLN-PHI and free PHI solution in diabetic rats (n = 6)

The oral bioavailability of ORLN-PHI was investigated in diabetic rats. The group treated with a subcutaneous injection of the same dose of free PHI was used as a control. The average plasma concentration of PHI over time in rats receiving equivalent treatments (60 μg/kg dose) is shown in Fig. 4. The pharmacokinetic parameters calculated using plasma PHI concentrations are shown in Table 2. As shown in Fig. 4, the levels of PHI rapidly increased within 1 h after subcutaneous administration of 60 μg/kg free PHI. The change in PHI concentration in the group orally administered with ORLN-PHI was slightly lower than that in the group subcutaneously injected with free PHI. In contrast, PHI was not observed in the plasma of rats treated orally with 60 μg/kg free PHI. The bioavailability of the group treated with ORLN-PHI relative to that of the group subcutaneously injected with free PHI was found to be 28.7%. These results demonstrate that ORLN-PHI could substantially improve the oral bioavailability of insulin. Furthermore, the half-life of oral ORLN-PHI was confirmed to be greater than that of the subcutaneously injected insulin, indicating the sustained efficacy of oral ORLN-PHI. The results of our pharmacokinetic analysis provide direct evidence for explaining the enhancement of oral insulin delivery by ORLN.

**Table 2:** Pharmacokinetic parameters of ORLN-PHI administered orally and PHI solution administered subcutaneously

Parameters	ORLN-PHI (ig)	PHI solution (s.c)
C_max_ (μIU/mL)	8.03 ± 2.68	15.49 ± 3.41
MRT (h)	3.5	2.7
AUC_0-8h_ ((μIU/mL) h)	25.29 ± 4.85	44.08 ± 3.74
F_rel_ (%)	28.7%

### Mechanism of action of ORLN-PHI on the enhancement of oral insulin delivery

#### In vitro release

**Fig. 5:**
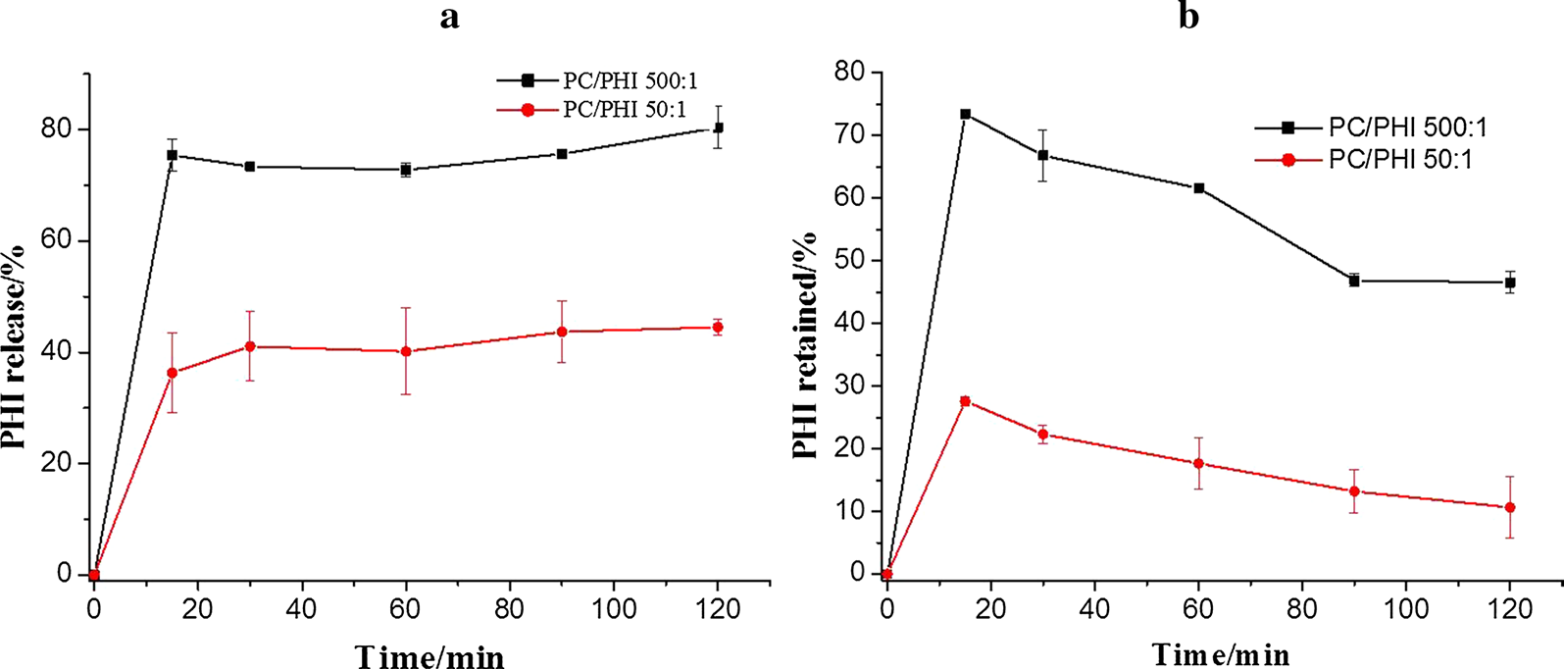
Results of **a** in vitro release and **b** enzymatic stability of ORLN-PHI formulation at 37 °C in artificial intestinal fluid as the release medium (pH 6.5). PHI was measured using high-performance liquid chromatography. Data are represented as the mean ± SD (n = 3)

The in vitro release profile for ORLN-PHI formulations is illustrated in Fig. 5a. When two ORLN-PHI formulations were placed in artificial intestinal fluid (pH 6.5) at 37 °C, the formulation with a higher PC content (PC/PHI 500:1) showed faster release than that with a lower PC content (PC/PHI 50:1). The proportion of PHI released from ORLN-PHI with a PC/PHI ratio of 500:1 was over 80% within 120 min. Furthermore, the proportion of PHI released from ORLN-PHI with PC/PHI ratio 50:1 was close to 40% after 120 min in the same medium. These results indicated that PC, which acted as a membrane-simulating material in ORLN, played a vital role in the release of PHI from oil solution. The mechanism underlying the enhancement of insulin release can be interpreted as follows: PC in ORLN improved the hydrophilic ability of the oil system, forcing water molecules to move to the oil solution when the ORLN formulation came into contact with the aqueous medium, thereby weakening the oil barrier of the ORLN preparations. We have thus clearly established that a higher PC concentration will lead to an increased release rate of PHI from ORLN-PHI. A guiding principle for the design of ORLN is that the PC content is associated with the release rate of ORLN formulations, especially for the delivery of macromolecules such as proteins and peptides.

#### In vitro enzymatic stability

Figure 5b shows the in vitro stability of PHI at 37 °C after placing two different formulations of ORLN-PHI with different PC contents in artificial intestinal fluid (pH 6.5) supplemented with trypsin. The proportion of remaining PHI was 46.5% and 10.6% for ORLN-PHI at a ratio of PC/PHI 500:1 and PC/PHI 50:1 in artificial intestinal fluid within 120 min, respectively. However, no native PHI was retained even for 5 min when free PHI solution was tested under the same conditions. From these results, it was found that PHI in ORLN with a higher PC content was more stable in trypsin. The protective effects of ORLN on insulin were thus exerted in a PC concentration-dependent manner.

#### In vitro insulin transport assay

**Fig. 6:**
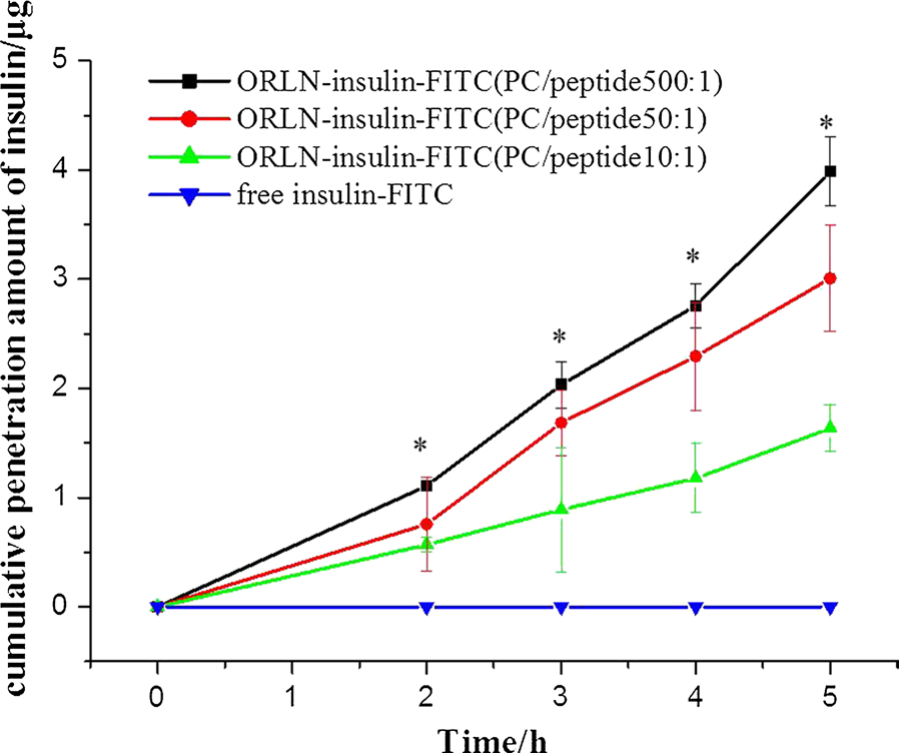
Enhancement of fluorescein isothiocyanate (FITC)-insulin transport across Caco-2 monolayers mediated by PC in the ORLN system. Data are represented as the mean ± SE (n = 3). *p < 0.05 vs free insulin treatment

**Fig. 7:**
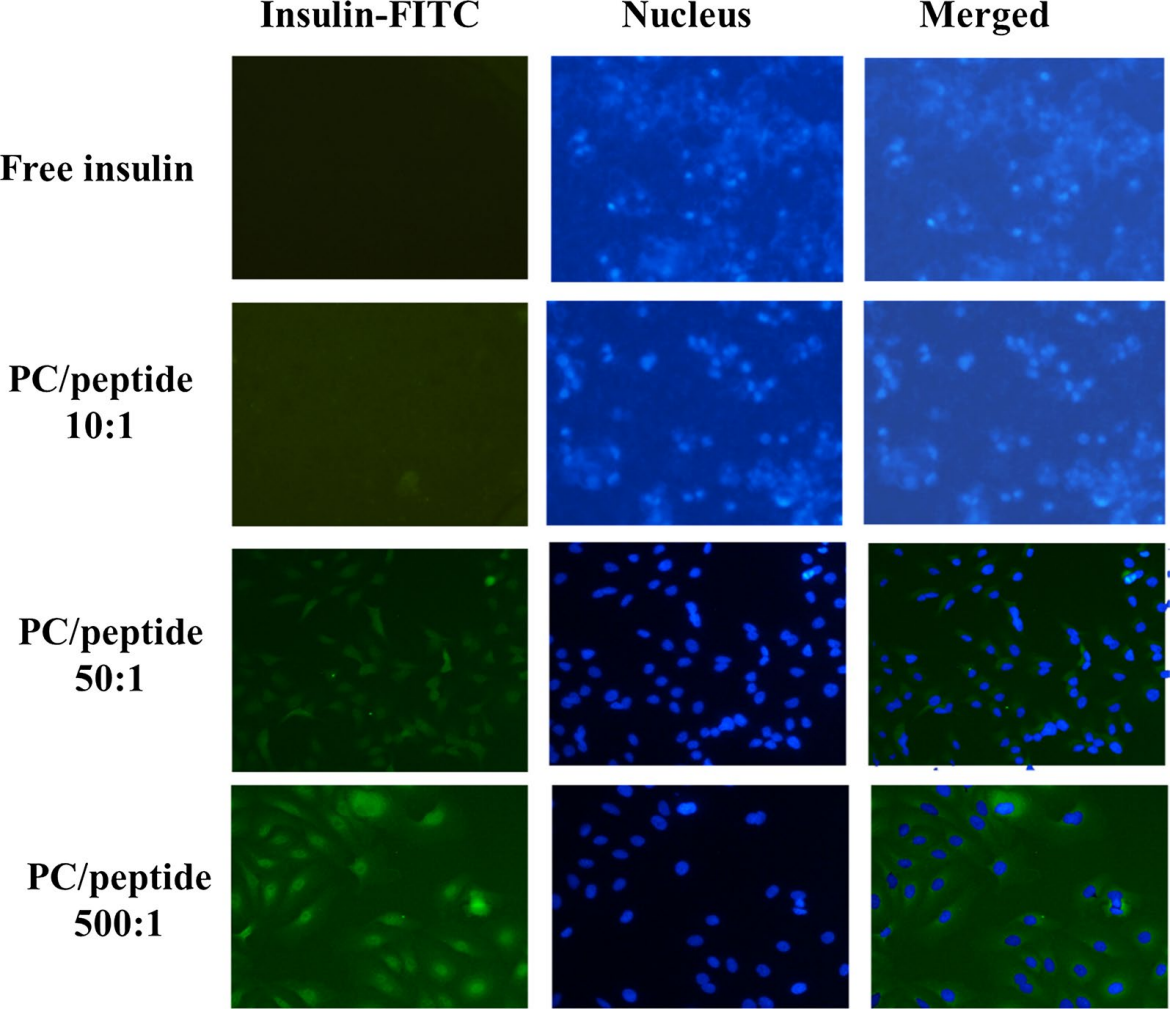
Representative fluorescence microscopy images of transwell membranes covered with a layer of Caco-2 cells and incubated for 5 h with ORLN-FITC-insulin containing various concentrations of PC. Images were taken at ×40 magnification. The images show DAPI-labeled nuclei (blue color), FITC-insulin (green color), and merged images of Caco-2 cells

As shown in Fig. 6, the ORLN system significantly improved the transport of FITC-insulin compared to free insulin. The enhancement of FITC-insulin transport by ORLN was also PC content-dependent. ORLN-FITC-insulin with a higher PC content (PC/insulin 500:1) increased insulin permeability by approximately four-fold across cell monolayers compared to ORLN-FITC-insulin with a lower PC content (PC/insulin 10:1). These results were confirmed through micrographic images of the transwell membranes at the end of the study after 5 h. The images clearly show increased FITC-insulin uptake of ORLN with higher PC concentrations by Caco-2 cells compared to that by control cells (Fig. 7). In addition, no decrease in TEER was observed during the experiments. When displaying the ORLN-PHI at the end of the experiment, FITC-dextran could not diffuse across the cell monolayers. The penetration of ORLN-PHI was likely mediated by the transcellular permeability of the insulin molecules across Caco-2 cells.

## Discussion

Oral administration of insulin must be able to overcome challenges such as the low pH of the GI tract, enzymatic degradation, and barriers including mucus layers and the intestinal epithelium [16]. The requirements for an effective drug carrier include preventing drug release in the stomach, reduced protease degradation, increased infiltration through the mucus layer, and successful permeation across the epithelium. Various approaches have been applied to design and prepare carriers for the oral delivery of insulin, including polymeric nanoparticles and hydrogels or other functional nanoparticles [17–20]. Most of these strategies have shown advantages in terms of the carriers themselves or been successful in animal experiments. Some nanoparticles are highly stable and prevent the release of drugs in an acidic environment through the modification of pH-sensitive polymers [21]. Other nanoparticles have been designed to target intestinal cells such as goblet cells to directly transport insulin [22]. However, the complexity of the synthesis of polymeric nanoparticles increases the manufacturing costs and restricts their applications in a clinical setting. Furthermore, these nanocarrier-related formulations show poor oral bioavailability.

In this study, the development of oral insulin using ORLN technology represents a significant advancement in insulin administration strategies. In contrast to conventional nanocarriers, liquid oil was used as a dispersing medium in this system and phospholipids were used as the shell material for the nanoparticles. Amphipathic phospholipid molecules can self-assemble into a regularly arranged internal polar region for hydrophilic molecules, while the non-polar tails radiate to the solvent. As a result, water-soluble proteins were encapsulated and formed a core–shell lipid nanoparticle in oil. The ORLN system combines the advantages of liposomes and lipid nanoparticles and avoids drawbacks such as poor stability and complicated drug-encapsulating processes. Unlike subcutaneously administered insulin, the effectiveness of ORLN-insulin was shown to be sustained until the end of the study at 17 h, demonstrating its potential for development as a long-acting oral insulin formulation.

The absorption-enhancing effect of ORLN on PHI was probably related to the decrease in enzymatic degradation of PHI in the intestinal tract, as well as the increase in drug transcytosis across the intestinal epithelia. The enhancement of insulin absorption by ORLN was attributed to the formulation of the PC shell and oil medium. Phospholipids played a dual role in absorption in the ORLN-PHI system, acting as both “helper” and “protector” for transcytosis under enzymatic conditions. Thus, a higher PC content led to increased hypoglycemic efficacy. When PHI was released from the oil phase into the aqueous medium, the molecules likely interacted with several external PC layers and to form vesicle-like structures in the intestinal fluid. This process may have decreased enzymatic degradation and increased the lipophilicity of PHI, thereby promoting absorption through the intestinal tract. An oil medium, such as MCT, can also work as a “helper” and “protector” for protein absorption because oil barriers can protect insulin from degradation by preventing direct contact with gastric acid or protease. Furthermore, the digestive products of oils, such as fatty acids, can promote oral absorption.

**Fig. 8:**
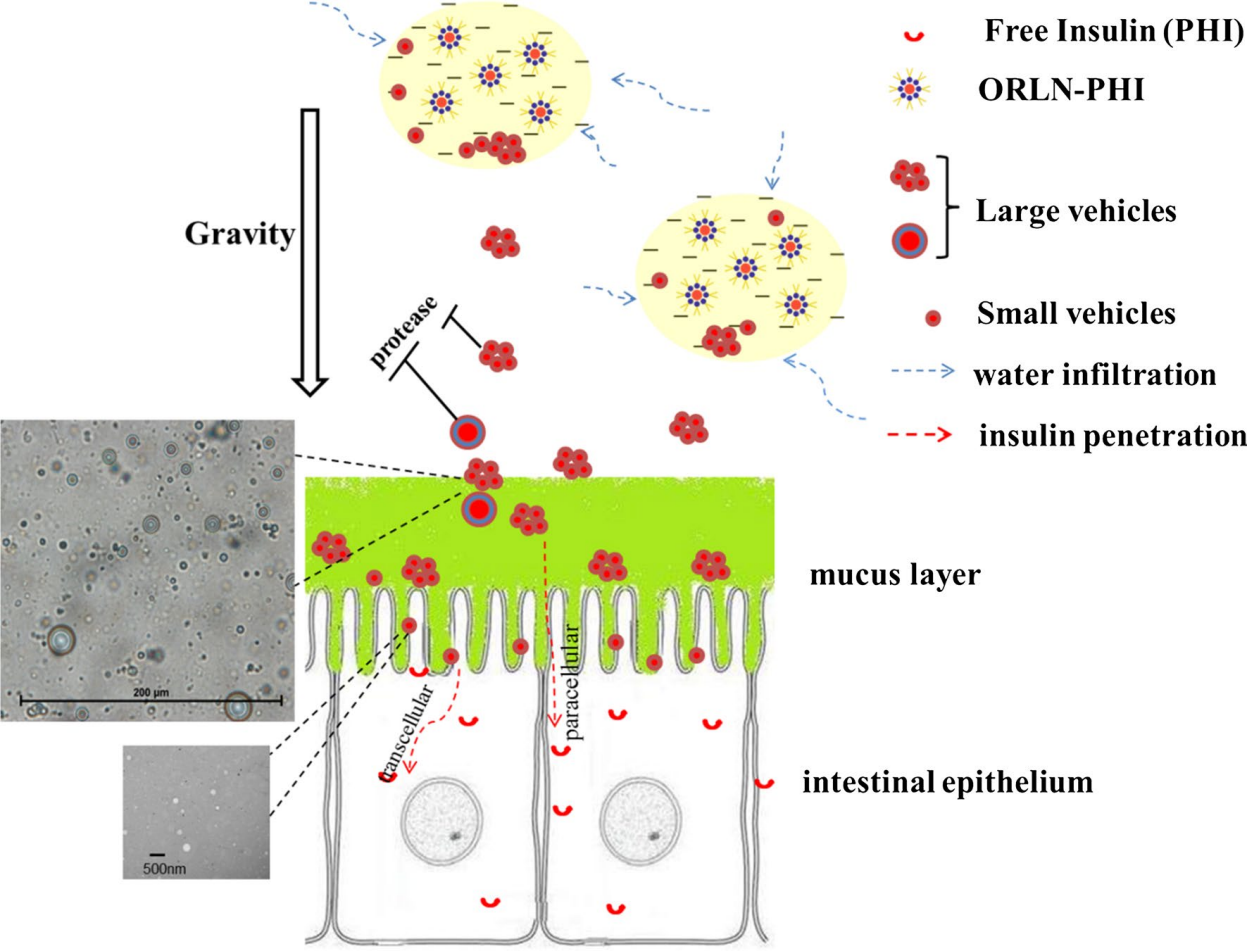
Mechanism of ORLN system in improving the intestinal absorption of insulin

The PC layer increased the hydrophilicity of the When ORLN oil solution mixed with an aqueous medium such as intestinal fluid, the movement of water molecules into the hydrophilic core of ORLN, which is dependent on the PC concentration in the system, can result in the reorganization of the reversed lipid nanoparticles and the formation of vesicles. Many nanoscale vesicles are known to aggregate to form larger micrometer-scale particles. These larger vesicles will enter the aqueous medium, driven by gravity and gastrointestinal motility, until they reach the surface of the intestinal epithelium. Finally, PC can interact weakly and non-covalently with insulin, increasing its lipophilicity and thereby its ability to cross the epithelium via a transcellular route (Fig. 8). PC acts as a water-absorbing agent and film material for the vesicles in this process. We investigated the mechanism of ORLN-PHI on the enhancement of oral insulin delivery. Our results showed that the enhancement was related to two effects of ORLN: a protective effect and transcellular enhancement. The oil barrier prevents the release of insulin into the stomach due to the absence of bile salts, thus avoiding chemical degradation in the acidic stomach environment. The formation of vesicles can decrease the contact between insulin molecules and intestinal fluid, thereby inhibiting enzymatic hydrolysis by proteases. Because PC served as a layer material to construct vesicles, the protective effects of ORLN on insulin were PC concentration-dependent.

The enhancement mechanism of PC was likely due to its weak and non-covalent interactions with insulin, which increased its lipophilicity and consequently its ability to cross the Caco-2 monolayers. PC layers also play a role in cell-membrane fusion with the intestinal epithelium, which is the driving force for transcellular insulin absorption. Moreover, the unchanged TEER value of the cell monolayers throughout the treatment indicated that insulin was transported transcellularly without any detectable alterations in the tight junctions between adjacent cells. The transcellular pathway of oral insulin implies that insulin can be safely transported without disturbing the integrity of the epithelium [23]. PC in ORLN played an important role in the absorption of insulin as a transcytosis “helper.” Thus, a higher PC content led to stronger hypoglycemic efficacy and higher oral bioavailability of the ORLN-insulin formulation. The enhancement mechanism of MCT is likely related to its digestive products, such as fatty acids. This amphipathic substance can play a similar role to that of PC in protein absorption.

## Conclusions

In this study, ORLN formulations were constructed to successfully encapsulate insulin. The ORLN system was confirmed to protect insulin molecules by forming PC vesicles in the intestinal fluid, thus avoiding direct contact between insulin and trypsin. Furthermore, PC played an important role in transcytosis at the intestinal wall. ORLN-PHI enhanced the oral absorption of insulin in rats compared to that observed for free PHI solution. The in vivo efficacy and pharmacokinetic parameters of the ORLN-PHI system showed encouraging results. The oral hypoglycemic effect of ORLN-PHI was significantly enhanced compared to that of free PHI. The oral bioavailability of ORLN-PHI was 28.7% relative to that of subcutaneously administered free PHI. Overall, ORLN shows potential as a promising nanocarrier for improving the oral absorption of insulin.

## Data Availability

All data generated or analyzed during this study are included in this published article.

## Abbreviations

ORLN: “Oil-soluble” reversed lipid nanoparticle
PC: phospholipid
PHI: peptide recombinant human insulin
